# Hendra Virus Outbreak with Novel Clinical Features, Australia

**DOI:** 10.3201/eid1602.090780

**Published:** 2010-02

**Authors:** Hume Field, Kylie Schaaf, Nina Kung, Craig Simon, David Waltisbuhl, Heather Hobert, Frederick Moore, Deborah Middleton, Allison Crook, Greg Smith, Peter Daniels, Ron Glanville, David Lovell

**Affiliations:** Biosecurity Queensland, Brisbane, Queensland, Australia (H. Field, N. Kung, D. Waltisbuhl, A. Crook, R. Glanville); Redlands Veterinary Clinic, Brisbane (K. Schaaf, C. Simon, H. Hobert, D. Lovell); Queensland Health, Brisbane (F. Moore, G. Smith); Australian Animal Health Laboratory, Geelong, Victoria, Australia (D. Middleton, P. Daniels)

**Keywords:** Hendra virus, horse, henipavirus, bats, viruses, zoonoses, Australia, dispatch

## Abstract

To determine the epidemiologic and clinical features of a 2008 outbreak of Hendra virus infection in a veterinary clinic in Australia, we investigated the equine case-series. Four of 5 infected horses died, as did 1 of 2 infected staff members. Clinical manifestation in horses was predominantly neurologic. Preclinical transmission appears likely.

Hendra virus (HeV) was first identified in 1994 after a fatal respiratory disease occurred in horses and humans in Australia (*1*,*2*). Fruit bats (*Pteropus* spp.) are the natural reservoir (*3*). Eleven bat-to-horse spillovers have now been identified, with 4 resulting in horse-to-human transmission. Infection in horses typically has been characterized by acute febrile illness with rapid, progressive respiratory system compromise and high case-fatality rates (*2*,*4*–*8*). A high case-fatality rate was again a feature of the 2008 outbreak, which we describe here: 4 of 5 infected horses died, as did 1 of 2 infected veterinary clinic staff. In contrast to previously reported cases, the clinical features of the infected animals and humans reflected primarily central nervous system involvement. We describe the equine case-series and discuss the epidemiologic features.

The outbreak occurred at an equine referral veterinary practice in Brisbane, Australia. Thirty-seven horses were residing on the premises (Figure 1). Five confirmed cases (all in horses admitted for unrelated illnesses) occurred from June 26 through July 24, 2008 (Appendix Table).

**Figure 1:**
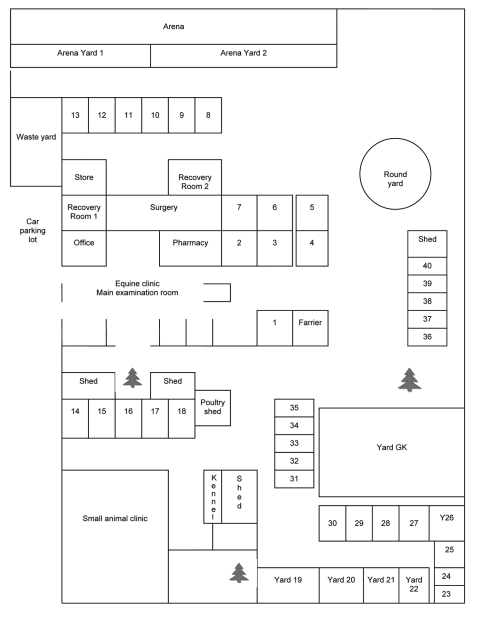
Layout of veterinary clinic where outbreak of Hendra virus infection occurred in horses, Australia, 2008. Individual horse stalls and yards are numbered 1–40. All yards are open, with yards 19–22 having a roofed shelter within.

## The Equine Case-Patients

### Case-Patient 1

Case-patient 1, a thoroughbred gelding 11 years of age, had resided at the clinic since June 27, 2007. On June 16, 2008, he was moved from yard GK to yard 19. On the morning of June 26, he showed ataxia, depression, disorientation, and extreme hypersensitivity when approached; the manifestations progressed to head tilting, left-side facial nerve paralysis, and endotoxemia. His condition deteriorated over several hours and he was euthanized (Table). A retrospective diagnosis of HeV infection was based on results of immunohistochemical testing on formalin-fixed cerebral tissue.

**Table:** Clinical laboratory and gross necropsy findings for confirmed equine cases of Hendra virus infection, Australia, 2008*

Case-patient no.	Hematologic/biochemical	Gross necropsy findings
1	Marked polycythemia, leukopenia, hyperkalemia, and hypochloremia; elevated levels of creatinine, bilirubin, globulins, and creatinine phosphokinase	Xanthochromic CSF
2†	Elevated bilirubin	
3	Elevated levels of globulins, creatinine phosphokinase, and fibrinogen; decreased bicarbonate	Xanthochromic CSF
4	NT	NT
5	NT	Unremarkable

*CSF, cerebrospinal fluid; NT, not tested**.** †Case-patient 2 recovered but was euthanized.

### Case-Patient 2

Case-patient 2, a thoroughbred gelding 2 years of age, was admitted on June 17 with bullous keratopathy of the right eye. He was placed in stall 2. On the afternoon of June 30, he was depressed, inappetant, and ataxic and had an elevated rectal temperature. Over the ensuing days, he exhibited a right-sided head tilt, facial nerve paralysis, unsteadiness, circling to the right, and intermittent recumbency. On July 4, he was asymptomatic. A July 7 blood sample yielded a titer of 2,048 in an HeV neutralization test. A blood sample obtained on July 10 yielded a titer of 4,096 and a positive real-time PCR result. He was moved to stall 24 on July 18. Serologic monitoring until he was euthanized on August 15 showed high titers by HeV neutralization test. A range of tissue specimens (liver, spleen, kidney, meninges, brain, spinal cord, and multiple lymph nodes), collected at necropsy, were positive by PCR and immunohistochemical testing.

### Case-Patient 3

Case-patient 3, a quarter horse gelding 5 years of age, was admitted on June 12 with conidiobolus nasal granulomas and placed in stall 40. Subsequently, he was moved to stall 16 (June 16–19), stall 17 (June 20–July 3), yard 26 (July 4), and stall 3 (July 5). On the evening of July 4, he was pyrexic and subdued. On July 5, he showed severe depression, ataxia, disorientation, and stranguria, and his condition progressed to recumbency. He was euthanized on the afternoon of July 5, and a necropsy was performed (Table). HeV was diagnosed on the basis of positive PCR results from nasal swabs and blood samples. Multiple tissues (meninges, brain, heart, and intestine) collected at necropsy were subsequently found to be positive by immunohistochemical testing. On the basis of the length of the known incubation period of HeV infection, we believe that case-patient 3 was likely housed in stall 17 at the time of exposure to infection.

### Case-Patient 4

Case-patient 4, a stock horse stallion 10 years of age, was admitted on May 15 with a fractured mandible. During June 17–24, he was in stall 1, and from June 25, in stall 4. On the morning of July 7, he was pyrexic, ataxic, severely depressed, disoriented, stranguric, and intermittently recumbent. Blood and urine specimens were positive for HeV by PCR. He was euthanized on July 8, and a limited necropsy was performed. Lung tissue was positive by PCR, and subsequently, by virus isolation. Case-patient 4 was most likely housed in stall 4 at the time of exposure to infection.

### Case-Patient 5

Case-patient 5, a stock horse mare 4 years of age, was admitted on June 24 with cutaneous tumors. She was placed in stall 5 until July 8, recovery room 2 until July 11, and then in stall 3. She exhibited a fever on July 23 and on July 24 was depressed, intermittently recumbent, and periodically pawing, head pressing, and leaning against the wall. A variety of tissues collected at necropsy were positive for HeV by PCR. The virus was subsequently isolated from kidney, spinal cord, spleen, and lymph node samples.

## Conclusions

The flagrant neurologic features in this outbreak strongly contrast with those of previous cases, in which acute respiratory disease predominated. However, mild neurologic signs were noted in several horses in 1994 (*4*), and nonrespiratory manifestations (colic, depression) also have been observed previously (*7*). Phylogenetic analyses indicate some genetic differences between the strain that caused this outbreak and previously identified strains (K. Halpin, pers. comm.), but experimental infection of horses with the outbreak strain produced both respiratory and neurologic signs (D. Middleton, unpub. data). Virus dose and route of infection could plausibly influence clinical features; alternatively, the neurologic predominance in this outbreak may simply indicate the spectrum of possible manifestations.

Patient records and epidemiologic evidence support our contention that case-patient 1 had the primary case. A roost of *Pteropus alecto* and *P. poliocephalus* bats, recognized reservoirs of Hendra virus (*9*), was located within 5 km of the practice, and bats were regularly observed in the immediate practice vicinity. The reported incubation period for HeV infection in horses is 4–16 days (*1*,*4*). We contend that case-patient 1 was exposed to infectious body fluids from a foraging bat (through contamination of pasture, feed, water, or yard rails) and that he was the originator of this outbreak. In the 4 days before his illness and euthanasia, case-patient 1 had been treated at the clinic for a minor laceration. The clinic was the operational hub of the veterinary practice and the evident focus of transmission in this outbreak. We believe that transmission to case-patients 2–4 followed contamination of surfaces or equipment by infectious body fluids (plausibly blood, urine, saliva, or nasal discharge) from case-patient 1 before onset of his clinical signs.

**Figure 2:**
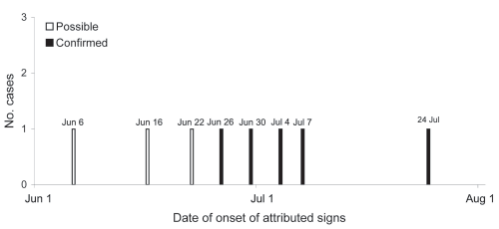
Epidemic curve of Hendra virus infection in horses, Australia, 2008. White bars represent the 3 possible cases; black bars represent the 5 confirmed cases.

Experimental studies suggest that horses may be infectious 48 hours before they show clinical signs (D. Middleton, unpub. data) At the time, case-patients 2–4 were housed in the clinic area or were being intensively treated, with daily or twice daily visits to the clinic, which would likely have increased their probability of becoming infected. Environmental swab samples from stalls 2, 3, and 4 yielded positive results by PCR, confirming that surfaces (walls, doors, wire mesh partitions between stalls) had been contaminated. Case-patient 5 had 3 possible sources of infection: most plausibly, case-patient 4, who had been in the adjacent stall until July 8; less plausibly, the recovered case-patient 2, who had been in stall 2 until July 18; and least plausibly, environmental contamination in stall 3 (occupied by case-patient 3 until July 5). The horse in yard 20, who had an extended opportunity for direct contact with case-patient 1, and the horses in stalls 6 and 7, who had extended opportunity for close contact with case-patients 2 and 5, respectively, did not acquire infection. These findings support previous observations that unassisted horse-to-horse transmission is inefficient. Three other horses died at the clinic without a definitive diagnosis before the death of case-patient 1, but the lack of clinical samples, conflicting epidemiologic evidence, and plausible alternative reasons for death precluded their inclusion as case-patients. However, they are considered possible case-patients (Figure 2).

Results of PCR and serologic tests for all other horses on the premises remained negative 4 weeks after case 5 occurred. No further cases have occurred.

Veterinarians and horse owners should consider HeV infection in any horse exhibiting acute-onset febrile illness, regardless of clinical manifestations, and implement appropriate risk assessment and management strategies. Biosecurity Queensland has compiled comprehensive veterinary guidelines (*10*)

## Addendum

Two additional spillover events have since occurred: in July–August 2009, 3 horses and 1 human were fatally infected (with a fourth horse nonfatally infected); and in August 2009, two horses were fatally infected.

## Supplementary Material

Appendix Table Location by date of confirmed case-patients with Hendra virus infection residing at an equine referral veterinary practice in Brisbane, Australia, 2008*
