# Errata—Vol. 16, No. 2

**DOI:** 10.3201/eid1604.C11604

**Published:** 2010-04

**Keywords:** Errata, Erratum, Correction

The article Hendra Virus Outbreak with Novel Clinical Features, Australia (H. Field et al.) contained several errors related to specific case descriptions and spillover events. The article has been corrected online http://wwwnc.cdc.gov/eid/article/16/2/09-0780_article.htm. 

