# Supplementary material for: Hendra Virus Outbreak with Novel Clinical Features, Australia
Source: Emerg Infect Dis. 2010 Feb;16(2):338–40. doi: 10.3201/eid1602.090780 (PMC2958006; doi:10.3201/eid1602.090780)
Supplement: Appendix Table — Location by date of confirmed case-patients with Hendra virus infection residing at an equine referral veterinary practice in Brisbane, Australia, 2008* [file 09-0780_appT-s1.pdf]

Appendix Table. Location by date of confirmed case-patients with Hendra virus infection residing at an equine referral veterinary practice in Brisbane, Australia, 2008*

Date	Stall/yard name/no.				
	Case-patient 1†	Case-patient 2	Case-patient 3	Case-patient 4‡	Case-patient 5
June					
10	GK			6	
11	GK			6	
12	GK		40	6	
13	GK		40	3	
14	GK		40	3	
15	GK		40	3	
16	19		16	3	
17	19	2	16	1	
18	19	2	16	1	
19	19	2	16	1	
20	19	2	17	1	
21	19	2	17	1	
22	19	2	17	1	
23	19	2	17	1	
24	19	2	17	1	5
25	19	2	17	4	5
26	**19**	2	17	4	5
27		2	17	4	5
28		2	17	4	5
29		2	17	4	5
30		**2**	17	4	5
July					
1		**2**	17	4	5
2		**2**	17	4	5
3		**2**	17	4	5
4		2	**26**	4	5
5		2	**3**	4	5
6		2		4	5
7		2		**4**	5
8		2		**4**	RR 2
9		2			RR 2
10		2			RR 2
11		2			RR 2
12		2			3
13		2			3
14		2			3
15		2			3
16		2			3
17		2			3
18		24			3
19		24			3
20		24			3
21		24			3
22		24			3
23		24			3
24		24§			**3**

*RR, recovery room. **Boldface** indicates dates on which clinical signs were observed.
†Long-term resident admitted on June 27, 2007.
‡Admitted on May 15, 2008.
§Remained in stall 24 after recovering until he was subsequently euthanized and a necropsy was performed on August 15, 2008.
